# Analysis of Phenolic and Flavonoid Contents, and the Anti-Oxidative Potential and Lipid Peroxidation Inhibitory Activity of Methanolic Extract of *Carissa opaca* Roots and Its Fractions in Different Solvents

**DOI:** 10.3390/antiox3040671

**Published:** 2014-10-20

**Authors:** Dildar Ahmed, Khaizran Fatima, Ramsha Saeed

**Affiliations:** Department of Chemistry, Forman Christian College (A Chartered University), Lahore-54600, Pakistan; E-Mails: khaizranfatima@yahoo.com (K.F.); ramsha.sd@gmail.com (R.S.)

**Keywords:** *Carissa opaca*, roots, flavonoids, phenolics, antioxidant

## Abstract

The objective of the present work was to investigate the anti-oxidative potential of methanolic extract of *Carissa opaca* roots and its fractions in solvents of different polarities. Total phenolic (TPC) and flavonoid (TFC) contents of methanolic extract were 211.95 ± 0.78 μg/mL gallic acid equivalents (GAE) and 8.35 ± 0.21 μg/mL rutin equivalents (RE), respectively. Ethyl acetate contained the highest amounts of both (TFC, 11.8 ± 0.28 RE; TPC, 342.80 ± 0.42 GAE) followed by chloroform fraction (TFC, 7.50 ± 0.14 RE; TPC, 275.85 ± 0.50 GAE). Extract and fractions displayed remarkable DPPH radical scavenging activity. EC_50_ values of methanolic extract was 0.88 mg/mL, while that of hexane, chloroform, ethyl acetate, *n*-butanolic and aqueous fractions were 0.58, 0.38, 0.29, 0.36 and 5.83 mg/mL, respectively, ethyl acetate fraction being most potent. The ethyl acetate fraction also showed the highest activity in terms of reducing power, phosphomolybdate and ABTS assays. All the fractions showed fairly good lipid peroxidation inhibitory activity, which remained almost constant over three days. Based on the results it can be concluded that roots of *Carissa opaca* contains phytochemicals with exploitable antioxidant, free radical scavenging, and lipid peroxidation inhibitory potential.

## 1. Introduction

Reactive oxygen and nitrogen species, abbreviated as ROS and RNS, respectively, are species with odd electrons called free radicals. They are very reactive groups of atoms and have potential to damage components of living cells when produced excessively [1,2]. They are continuously produced in the human body but are simultaneously destroyed by its antioxidant defense mechanism. Continuous exposure to free radicals can contribute to fatal ailments including cancer, cardiovascular and neurodegenerative diseases, and age related disorders [3,4]. As plants constitute a virtually unending source of bioactive natural products, they are the subject of extensive research worldwide with the aim to explore novel therapeutic agents.

*Carissa opaca* (family Apocynaceae) is an evergreen, thorny shrub found in the Himalayan areas of Indo-Pakistan subcontinent [5,6,7]. The plant has a number of ethno-medicinal applications and all of its parts including leaves, fruit and roots are considered to be useful in various diseases [8,9]. Its leaves are used to cure fever, jaundice, hepatitis and asthma [10,11]. Its fruit is considered to be an aphrodisiac [12,13]. Roots of the plant are known to be purgative, and have an ability to heal wounds and injuries [14]. Ground roots are mixed with pericarp of mango in water and used as wormicide of the intestine [15]. The chemical constituents found in *C. opaca* include flavonoids, terpenoids, coumarins, glycosides, tannins and anthroquinones [16]. Antioxidant activities of both fruit and leaves of *C. opaca* have been studied under different conditions [16,17,18].

In continuation of our research on *C. opaca* [17,19], we investigated, in the present work, its roots for total phenolic and flavonoid contents, antioxidant and free radical scavenging properties and lipid peroxidation inhibitory activity. As far as we could explore, this is the first study of its type on the roots of *C. opaca*.

## 2. Experimental Section

### 2.1. Chemical

All the chemicals used in the present study were of analytical grade. Ammonium molybdate, sodium dihydrogen phosphate, disodium hydrogen phosphate, potassium ferricyanide, ammonium thiocyanate, potassium persulfate sodium nitrite and gallic acid were purchased from Riedel-de-Haen (Seelze, Germany). Tween 20 and ascorbic acid were bought from Fischer Chemicals (Loughborough, UK). TPTZ (2,4,6 tripyridyl-*s*-triazine), trichloroacetic acid, ABTS (2,2′-azino-bis(3-ethylbenzothiazoline-6-sulphonic acid), Trolox (6-hydroxy-2,5,7,8-tetramethylchroman-2-carboxylic acid), DPPH (2,2-diphenyl-1-picrylhydrazyl) were purchased from Sigma-Aldrich (St. Louis, MO, USA). Rutin was purchased from Alfa Aesar (Karlsruhe, Germany), Folin-Ciocalteu reagent from Merck (Darmstadt, Germany), and linoleic acid from Bio-World (Dublin, OH, USA). Solvent used for extraction and fractionation were of HPLC grade.

### 2.2. Collection and Preparation of Plant Material

#### 2.2.1. Collection of Plant Material

Roots of *C. opaca* were collected from hills of Abbottabad (Pakistan) in March 2013. The identification of the plant was confirmed by the taxonomist Mr. Ajaib Khan of GC University, Lahore, Pakistan (voucher specimen: GC-Herb Bot 2271). The roots were separated from the aerial parts of the plants, washed with distilled water and allowed to air dry in shade. They were crushed and ground to obtain finely divided powder.

#### 2.2.2. Extraction and Fractionation

A methanolic extract of the powder was obtained through cold maceration method by soaking the material in pure methanol for 15 days followed by filtration. The process was repeated thrice and the extracts were combined. The solvent was evaporated on rotary evaporator to obtain dried crude methanolic extract. The methanolic extract was then suspended in distilled water in a separatory funnel and partitioned successively with hexane, chloroform, ethyl acetate, and 1-butanol to obtained fractions in these solvents. This process left residual aqueous fraction at the end. The solvents were removed with a rotary evaporator at low pressure (about 10 mbar) to produce dried fractions.

### 2.3. Determination of Total Phenolic Content (TPC)

Colorimetric protocol [20] was used to determine total phenolic content of methanolic extract of *C. opaca* roots and its fractions. In a test tube, 40 μL (1 mg per 1 mL of methanol) of the plant extract (or standard gallic acid solution), 3.16 mL distilled water and 200 μL Folin-Ciocalteu reagent were put and mixed by shaking gently. After an incubation of 8 min, 600 μL sodium carbonate solution was added and mixed. The mixture was incubated at 40 °C for 30 min before recording its absorbance in a spectrophotometer at 765 nm against a blank. The blank contained 40 μL methanol in place of sample. Gallic acid was used as a standard. Its calibration curve was drawn and the total phenolic content was expressed as micrograms per milliliter of gallic acid equivalents (μg/mL of GAE).

### 2.4. Determination of Total Flavonoid Content (TFC)

Total flavonoid contents of plant samples were determined using aluminum chloride colorimetric method [21]. Briefly, 1 mg of plant extract was dissolved in 1 mL of methanol. In a test tube, 0.3 mL (1 mg/mL in methanol) of plant sample (or standard rutin solution) and 3.4 mL 30% aqueous methanol, 150 μL sodium nitrite solution (0.5 M) and 150 μL aluminum chloride solution (0.3 M) was mixed. After an interval of 5 min, 1 mL NaOH solution (1 M) was added. The absorbance was measured at 506 nm against a blank. The blank contained an equal amount of methanol in place of the sample. Rutin was used as a standard. Its calibration curve was drawn and total flavonoid content of a plant sample was expressed a μg/mL of rutin equivalents (RE).

### 2.5. DPPH Radical Scavenging Assay

Free radical scavenging activities of the methanolic extracts of *C. opaca* roots and its fractions were determined according to the DPPH methods of Ahmed *et al.* and Brand-Williams *et al.* [21,22]. The stock solution of DPPH radical (24 mg/100 mL in methanol) was prepared. By diluting this solution with methanol, a working solution was prepared to obtain an absorbance of 0.980 ± 0.02 at 517 nm. Stock solution of plant extract/fraction was prepared in methanol with concentration 4 mg/mL. From this, different dilutions (0.2–2.0 mg/mL) were made. The test mixture contained 3 mL DPPH working solution and 100 μL of a sample. The mixture was incubated at 37 °C for 30 min in dark. Absorbance of each sample was recorded at 517 nm. For a negative control, 100 μL of methanol was added in place of plant sample. Ascorbic acid was used as a positive control. Inhibition curves were made and IC_50_ value were calculated for all samples. The free radical scavenging activity of each sample was calculated by using the formula:

% Scavenging Activity = 100[(*Ac* – *As*)/*Ac*] (1)

where *Ac* and *As* are absorbances of negative control and sample, respectively.

### 2.6. ABTS Radical Scavenging Assay

ABTS (2,2′-azinobis(3-ethylbenzothiazoline)-6-sulphonic acid) assay was conducted by protocol of Re *et al.* [23]. ABTS (38.36 mg) was dissolved in distilled water and the volume was made up to 9.5 mL, and 245 μL 100 mM potassium persulfate was added. The volume was made up to 10 mL by distilled water to give the final concentration of ABTS stock solution as 7 mM. The stock solution was kept in dark for 18 h at room temperature. To prepare the ABTS working solution, ABTS stock solution was diluted with PBS buffer pH (7.4) to an absorbance of 0.7 ± 0.02). In a test tube, 2.99 mL ABTS working solution was added and mixed with 10 μL (4 mg per mL of methanol) of the plant extract solution (or standard). A negative control was prepared by adding 10 μL of methanol in place of sample. The absorbance was measured at 734 nm for eight min with the interval of 30 seconds and percentage free radical scavenging activity as per this assay was calculated by using the formula:

% Activity = 100(1 − Sample Absorbance/Control Absorbance)
(2)

Gallic acid solution (0.5 mg /mL of distilled water) and ascorbic acid solution (0.5 mg/mL of distilled water) were used as positive control. The ABTS radical scavenging activity was also determined in terms of Trolox equivalents. Trolox stock solution was prepared by dissolving 7.5 mg in 10 mL PBS. The volume was made to 20 mL to give the concentration of 1500 μM. From this stock solution, dilutions with concentrations of 1250 μM, 1000 μM, 750 μM, 500 μM, 250 μM, 100 μM and 50 μM were made by mixing 2.5 mL, 2 mL, 1.5 mL, 1 mL 0.5 mL, 0.2 mL and 0.1 mL of stock solution in distilled water to the volume of 3 mL.

### 2.7. Reducing Power Assay

This assay was performed as per the protocol reported by Oyaizu *et al.* [24]. In a test tube, 2.5 mL (1 mg/mL) of the plant extract (or standard gallic acid solution) was taken and mixed with 2.5 mL sodium phosphate buffer (0.2 M, pH 6.6) and 2.5 mL potassium ferricyanide solution (1%). The mixture was incubated at 50 °C for 20 min. After incubation, 2.5 mL trichloroacetic acid (10%) was added and the solution was centrifuged at 650 rpm for 10 min. Then, 5 mL supernatant was mixed with 5 mL distilled water and 1 mL ferric chloride solution (0.1%) was added. The blank had 2.5 mL methanol instead plant sample. The absorbance was measured at 700 nm.

### 2.8. Phosphomolybdate Assay

Total antioxidant capacity of the methanolic extracts *Carissa opaca* roots was determined as per the reported method [20,25]. In a test tube, 300 μL sample (0.3 mg/mL in methanol) was mixed with 3 mL phosphomolybdate reagent (0.6 M sulfuric acid, 28 mM sodium phosphate and 4 mM ammonium molybdate) and the test tube was capped with silver foil and incubated in water bath at 95 °C for 90 min. The mixture was cooled to room temperature. Its absorbance was measured at 765 nm against a blank. For blank, 300 μL methanol was added in place of plant sample. Ascorbic acid was used as a standard and the results were expressed as μg/mL of ascorbic acid equivalents.

### 2.9. Lipid peroxidation Inhibitory Assay

A reported protocol [26,27] was used to determine lipid peroxidation inhibitory activity of methanolic extract of *Carissa opaca* roots and its fractions. In a test tube, 100 μL (5 mg per mL of methanol) of plant extract (or standard BHA) was mixed with 2.4 mL potassium phosphate buffer (pH 7) and 2.5 mL linoleic acid emulsion. The mixture was incubated at 37 °C for 25 min. Then, 100 μL of this mixture was regularly taken at 24 h intervals for 120 h and dissolved in 3.7 mL ethanol. It was reacted with 100 μL of ferrous chloride (20 mM) and then 100 μL ammonium thiocyanate (30%) was added. Absorbance was measured at 500 nm. Control consisted of 2.4 mL potassium phosphate buffer, 2.5 mL of linoleic acid emulsion and 100 μL methanol. Linoleic acid emulsion was prepared by mixing 175 μL Tween 20 (emulsifier) and 155 μL of linoleic acid and the volume was made to 50 mL by adding potassium phosphate buffer (pH 7).

### 2.10. Statistical Analysis

All the experiments were carried out at least three times (*n* = 3) and statistical mean was calculated ± SD using Excel 2010 (Microsoft Corporation, Redmond, WA, USA). The same program was used to calculate EC_50_ values of DPPH assay.

## 3. Results and Discussion

Methanolic extract of *C. opaca* roots and its fractions in hexane, chloroform, ethyl acetate, 1-butanol and water were subjected to different antioxidant assays.

### 3.1. Total Flavonoid and Total Phenolic Contents

Total flavonoid and phenolic contents of methanolic extract of *C. opaca* roots and its fractions were determined and results are displayed in Table 1.

**Table 1:** Total flavonoid content (TFC) and total phenolic content (TPC) of methanolic extract of *Carissa opaca* roots and its fractions in various solvents, and their EC_50_ values in DPPH assay (*n* = 3)*.*

Extract/Fraction	TFC, RE (μg/mL)	TPC, GAE (μg/mL)	EC_50_ (mg/mL)
**Methanolic**	8.35 ± 0.21	211.95 ± 0.78	0.877 ± 0.002
**Hexane**	nd *	111.25 ± 0.35	0.584 ± 0.004
**Chloroform**	7.50 ± 0.14	275.85 ± 0.50	0.380 ± 0.002
**Ethyl acetate**	11.8 ± 0.28	342.80 ± 0.42	0.290 ± 0.005
***n*-Butanolic**	4.85 ± 0.07	194.70 ± 0.57	0.360 ± 0.012
**Aqueous**	5.30 ± 0.14	73.85 ± 0.50	5.830 ± 0.007

* Mixture was turbid and there was no change in color. RE, rutin equivalents; GAE, gallic acid equivalents.

The methanolic extract of the roots was found to contain considerable quantities of polyphenols and flavonoids as evidenced by its total phenolic (TPC) and flavonoid (TFC) contents, which were 211.95 ± 0.78 μg/mL gallic acid equivalents (GAE) and 8.35 ± 0.21 μg/mL rutin equivalents (RE), respectively. Ethyl acetate contained highest amounts of both (TFC, 11.8 ± 0.28 RE; TPC, 342.80 ± 0.42 GAE) followed by chloroform fraction (TFC, 7.50 ± 0.14 RE; TPC, 275.85 ± 0.50 GAE). Order of TPC among fractions was: ethyl acetate > chloroform > *n*-butanolic > hexane > aqueous. Order of TFC was almost same except the aqueous fraction showed slightly higher value than *n*-butanolic.

### 3.2. DPPH Free Radical Scavenging Activity

The antioxidant or free radical scavenging activities of methanolic extract of *C. opaca* roots and its fractions were determined as a function of concentration and results are displayed in Table 1 in terms of EC_50_.

The results revealed that roots of *C. opaca* had significant anti-oxidative potential. Ethyl acetate fraction was most active with EC_50_ of 0.29 mg/mL. Chloroform and *n*-butanolic fractions showed almost equal activity, 0.38 and 0.36 mg/mL, respectively. Aqueous fraction was least active (5.83 mg/mL). Order of activity among fractions was: ethyl acetate > chloroform > *n*-butanolic > hexane.

Although both electron transfer (ET) and hydrogen transfer (HT) pathways have been proposed for DPPH action, HT seems to predominate [28]. This assay, therefore, measures the ability of a sample to donate H radical to DPPH radical. Phenols and flavonoids have been found to be powerful free radical scavengers. The results of this assay indicated that roots of *C. opaca* have good quantities of compounds capable of free radical scavenging.

### 3.3. ABTS Radical Scavenging Activity

**Figure 1:**
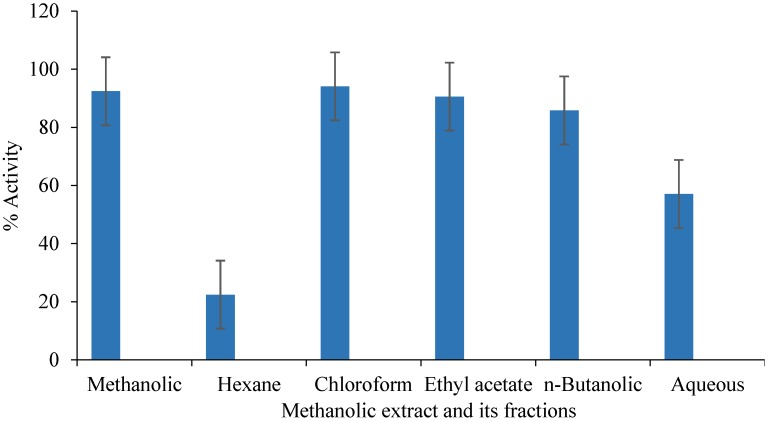
Percent free radical scavenging activity of methanolic extract of *Carissa opaca* roots and its fractions in ABTS assay (*n* = 3); Concentration of each sample was 1 mg/mL.

**Figure 2:**
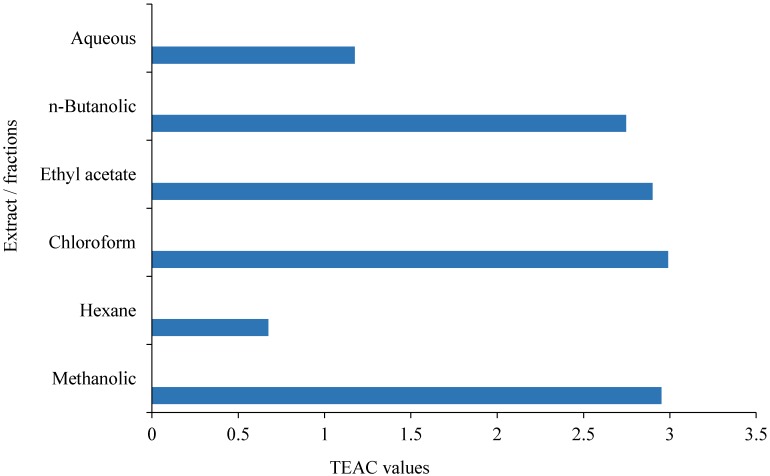
ABTS radical scavenging activity methanolic extract of *Carissa opaca* roots and its factions in terms of TEAC (Trolox equivalent antioxidant capacity) (*n* = 3).

Free radical scavenging activity of methanolic extract of *C. opaca* roots and its fractions were determined according to ABTS radical assay and results are displayed in Figure 1. The activity was also determined in terms of TEAC (Trolox equivalent antioxidant capacity) values (Figure 2). TEAC measures the antioxidant capacity of a sample as compared to Trolox (6-hydroxy-2,5,7,8-tetramethylchroman-2-carboxylic acid), a known antioxidant. Methanolic extract exhibited remarkable activity in this assay. Chloroform and ethyl acetate fractions were highly active and showed comparable potency. At concentration of 1 mg/mL, their activities were (%) 94.11 ± 0.001 and 90.58 ± 0.012, respectively. Hexane fraction was least active with only about 22% activity at concentration 1 mg/mL followed by aqueous fraction. *n*-Butanolic fraction showed moderate activity. Order of activity among fractions was: chloroform > ethyl acetate > *n*-butanolic > aqueous > hexane. The TEAC values were also in the same order.

There is almost complete correspondence between DPPH and ABTS radical scavenging activities. Ethyl acetate fraction, however, showed slightly higher activity in the DPPH assay than chloroform fraction.

### 3.4. Antioxidant Activities as per Reducing Power Assay

**Figure 3:**
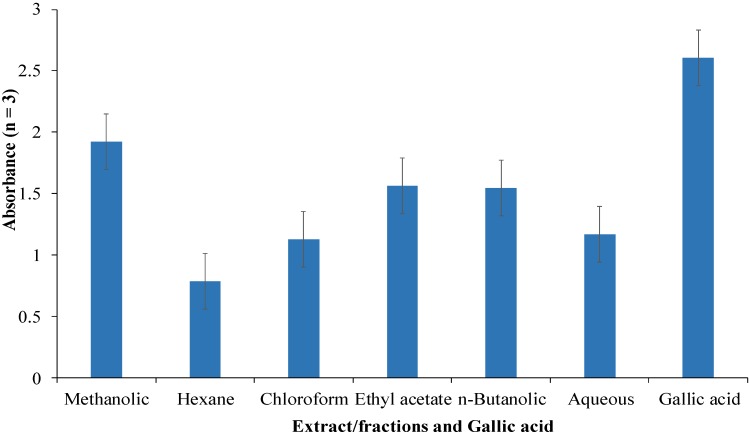
Antioxidant activities as per reducing power assay (mean absorbance at 700 nm) of methanolic extract of *Carissa opaca* roots and its fractions in various solvents (*n* = 3).

Antioxidant activities of methanolic extract of *C. opaca* roots and its fractions were determined as per reducing power assay and results are shown in Figure 3. The results are in the form of mean absorbance (*n* = 3), which is a direct measure of antioxidant activity of a sample. Ethyl acetate and *n*-butanolic factions showed almost equal antioxidant activity in this assay. They were most potent among all the fractions. Chloroform and aqueous fractions also showed good activity. The hexane fraction, however, did not exhibit appreciable activity. This is an electron transfer (ET) assay and measures the ability of a sample to donate an electron to destroy a free radical. The order of activity among fractions was: ethyl acetate > *n*-butanolic > aqueous > chloroform > hexane.

### 3.5. Antioxidant Capacity as per Phosphomolybdate Assay

**Figure 4:**
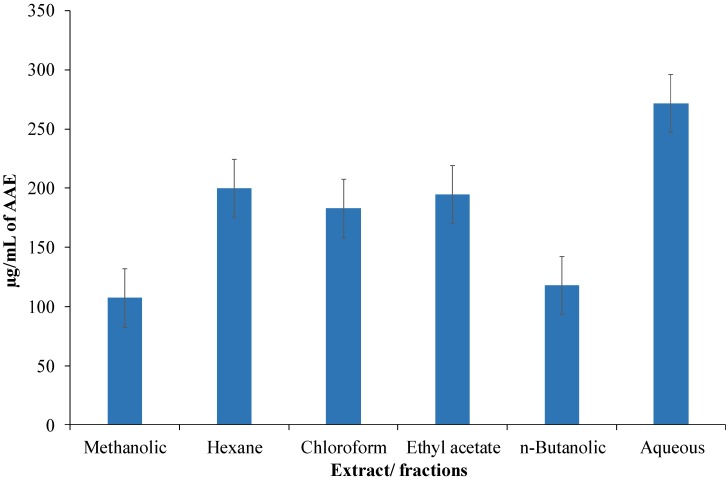
Antioxidant capacity of methanolic extract of *Carissa opaca* roots and its fractions in various solvents as per phosphomolybdate assay in units of μg/mL of ascorbic acid equivalents (AAE).

Antioxidant capacity of methanolic extract of *C. opaca* roots and its fractions was determined as per phosphomolybdate assay and results are shown in Figure 4. The results are in terms of mg/mL of ascorbic acid equivalents (AAE). Results showed an interesting trend. While all the samples displayed remarkable activity, the most polar aqueous (271.6 ± 0.0023 mg/mL AAE) fraction exhibited the highest activity followed by the most non-polar hexane fraction (199.96 ± 0.011 mg/mL AAE). This suggested that roots of this plant have a broad range of compounds having ability to scavenge free radicals.

### 3.6. Lipid Peroxidation Inhibitory Activity

**Figure 5:**
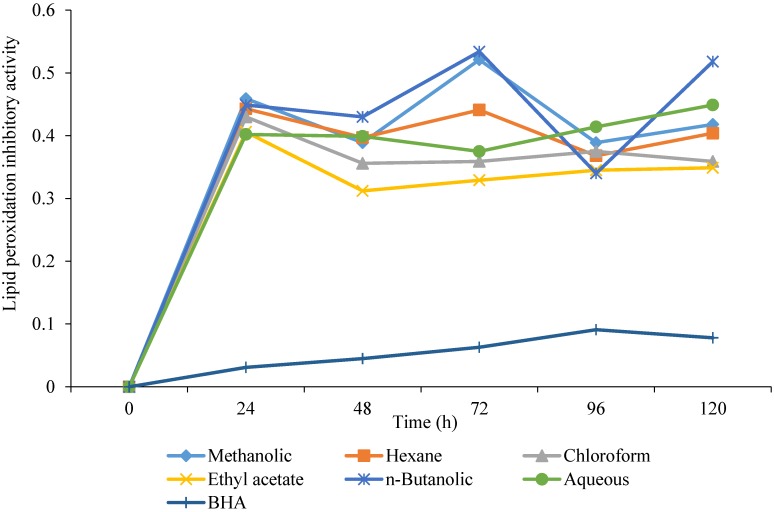
Lipid peroxidation inhibitory activity of methanolic extract of *Carissa opaca* roots and its fractions in various solvents and BHA (*n* = 3).

Lipid peroxidation inhibitory activity of methanolic extract of *C. opaca* roots and its fractions in different solvents were determined over a period of 120 h and results are displayed in Figure 5. The methanolic extract and its fractions showed fairly good lipid peroxidation inhibitory activity, which remained almost constant over three days. As a whole, the ethyl acetate fraction proved to be the most potent and consistent inhibitor of lipid peroxidation followed by chloroform fraction.

Roots of *C. opaca* exhibited, in assays discussed above, quite considerable antioxidant activity. There was good correlation between total phenolics and flavonoids, and between these polyphenols and antioxidant activities found through different assays. In general, moderately polar fractions, *i.e.*, ethyl acetate and chloroform, displayed the highest activity. Hexane fraction proved to be least potent, while *n*-butanolic and aqueous fraction exhibited moderate potency. The trend is in harmony with the results of many other studies. The ethyl acetate fraction, for example, has generally been noted to be the most potent antioxidant [4,29]. This trend may be attributed to the excellent ability of ethyl acetate to dissolve flavonoids and other polyphenols in it [30,31]. Phenolics constitute a class of diverse phytochemicals which are among the most potent antioxidants [32]. Investigation into ethyl acetate and chloroform fractions should yield individual chemical compounds responsible for most of their antioxidant potential.

## 4. Conclusions

Natural antioxidants are needed for their therapeutic and “nutraceutical” applications. Roots of *Carissa opaca* possessed remarkable antioxidant activity. Use of crude extracts can be pharmacologically useful. However, isolation and characterization of phytochemicals will help to establish structure-activity relationships in the discovery of new drugs. In-depth phytochemical investigation of the highly active ethyl acetate and chloroform fractions is especially recommended.
